# Real World Posaconazole Pharmacokinetic Data in Paediatric Stem Cell Transplant Recipients

**DOI:** 10.3390/children12040467

**Published:** 2025-04-05

**Authors:** Csaba Kassa, Katalin Csordás, Lídia Hau, Orsolya Horváth, Krisztián Kállay, Gabriella Kertész, Márton Kiss, János Sinkó, Ágnes Wolfort, Gergely Kriván

**Affiliations:** 1 Department of Pediatric Hematology and Stem Cell Transplantation, National Institute of Hematology and Infectious Diseases, South-Pest Central Hospital, 1097 Budapest, Hungary; csordas.katalin@semmelweis.hu (K.C.); kallay@dpckorhaz.hu (K.K.); kertesz.gabriella@dpckorhaz.hu (G.K.); krivan.gergely@dpckorhaz.hu (G.K.); 2 Pediatric Center, Semmelweis University, 1085 Budapest, Hungary; horvath.orsolya@semmelweis.hu; 3 Independent Researcher, 1132 Budapest, Hungary; 4 Heim Pál Children’s Hospital, 1089 Budapest, Hungary; janos.sinko@dpckorhaz.hu; 5 Department of Hematology and Stem Cell Transplantation, National Institute of Hematology and Infectious Diseases, South-Pest Central Hospital, 1097 Budapest, Hungary; agi.wolfort@gmail.com

**Keywords:** posaconazole, therapeutic drug monitoring, stem cell transplantation, paediatric, breakthrough infection

## Abstract

**Background:** Invasive fungal disease is a significant cause of morbidity and mortality in allogeneic hematopoietic stem cell transplant (alloHSCT) recipients. Posaconazole, a broad-spectrum triazole, is widely used as prophylaxis. **Methods:** We conducted a monocentric, retrospective study to present real-world data on posaconazole trough levels in paediatric alloHSCT patients. The main objective was to determine the required daily dose of posaconazole in paediatric patients. We analysed factors influencing posaconazole levels, and the association between posaconazole levels and breakthrough fungal infection. **Results:** Among 102 allogeneic HSCT recipients, we measured posaconazole plasma concentrations in 548 blood samples. The required daily doses to reach a target range of 0.7–2.0 mg/L were 15.22 (suspension), 7.52 (tablet), and 7.84 mg/kg (intravenous). Patients aged < 13 years needed higher doses to achieve the target range. The presence of enteral symptoms during prophylaxis was associated with lower plasma concentrations (*p* < 0.001), while co-administration of proton pump inhibitors did not (*p* = 0.09). Eight breakthrough infections occurred; low levels of posaconazole (<0.7 mg/L) were observed in five out of eight cases. The Cox regression model showed that higher mean plasma concentrations decreased the hazard of breakthrough infections. **Conclusions:** The tablet and intravenous formulations of posaconazole outperformed the suspension in terms of predictability. Our analyses on breakthrough infections and posaconazole plasma levels suggest an exposure–response relationship.

## 1. Introduction

Invasive fungal disease (IFD) is a significant cause of morbidity and mortality in patients undergoing allogeneic hematopoietic stem cell transplantation (allo-HSCT). As in adult patients, the most important risk factors in the paediatric setting are prolonged neutropenia, acute or chronic graft-versus-host disease (GvHD) and high-dose corticosteroid exposure [1]. The incidence of proven and probable IFDs after paediatric allo-HSCT varies between 12–15% under different antifungal prophylaxis regimens [2,3,4].

Posaconazole, a broad-spectrum triazole antifungal agent, is approved for prophylaxis of IFD in high-risk patients aged ≥ 2 years. To date, there are four different drug formulations: posaconazole oral suspension (POS), delayed-release tablet (DRT), intravenous (IV), and powder for oral suspension (PFS). POS was approved first, and is generally well tolerated and easy to administer. However, absorption is strongly influenced by meals, beverages, diarrhoea, and gastric pH, leading to some practical limitations and the need for therapeutic drug monitoring (TDM) [5,6]. These problems can be overcome with the use of DRT and, in particular, IV dosing, providing more consistent, once-daily dosing for paediatric patients [7,8]. In 2021, a novel PFS formulation was approved by the FDA. Groll and colleagues conducted an open-label, sequential dose-escalation study in paediatric patients, suggesting that by the once-daily administration of 6 mg/kg PFS, a target exposure required for effective antifungal prophylaxis can be achieved [9]. Thus, PFS may combine the advantages of both POS and DRT formulations.

TDM is recommended in patients receiving triazole-based therapy for invasive aspergillosis (IA), prolonged azole prophylaxis, or other therapies expected to interact with azoles to avoid treatment failure or toxicity due to suboptimal or excessive drug exposure [10,11]. In the paediatric setting, there are limited data on the optimal dosing of various posaconazole formulations, underscoring the importance of TDM in this population. Accordingly, the ECIL-8 guideline for the diagnosis, prevention, and treatment of invasive fungal diseases in paediatric patients with cancer or after hematopoietic cell transplantation suggests TDM for all children on posaconazole prophylaxis [12].

While most experts agree on the need for posaconazole TDM, determining an optimal target trough concentration (C_min_) for prophylaxis and treatment continues to generate much debate. An early clinical pharmacokinetic analysis showed that higher plasma concentrations were associated with a higher response rate [13]. So far, trough levels of 0.5–0.7 mg/L are usually accepted for prophylaxis and 1.0–1.25 mg/L for therapy [14,15]. These cut-off values were derived from studies involving patients of different ages, with different indications, and from different geographic areas without regard to the sensitivity of the relevant fungal pathogens. These values, however, may not be relevant to paediatric patients undergoing allo-HSCT. The authors point out that optimal drug exposure is necessary not only for treatment success but also to prevent the emergence of resistant strains [16].

Here, we present real-world data on posaconazole trough levels in paediatric patients undergoing allogeneic HSCT.

## 2. Materials and Methods

### 2.1. Setting

We conducted a monocentric, retrospective, cross-sectional study at South-Pest Central Hospital, National Institute of Hematology and Infectious Diseases, Budapest, Hungary. Paediatric patients (age < 18 years old) who underwent allo-HSCT and received posaconazole prophylaxis between 01/OCT/2012 and 30/SEP/2019 were included. We collected medical data (e.g., transplant and clinical parameters, and posaconazole levels) from source documents. The study was conducted with the approval of the Institutional Review Board (16185-002/2022).

### 2.2. Objectives

We aimed to provide real-world pharmacokinetic data from paediatric patients on posaconazole prophylaxis undergoing allo-HSCT. The primary objective of this study was to determine the daily dose required to achieve the target level between 0.7 and 2.0 mg/L with three different formulations of posaconazole (POS, DRT, IV) in paediatric patients.

Secondary objectives were to describe the influence of enteric symptoms (diarrhoea and/or vomiting), concomitant use of proton pump inhibitors, and mucositis on posaconazole serum levels. In addition, we aimed to analyse the association between posaconazole serum levels, breakthrough fungal infection, and all-cause mortality at day 30 after prophylaxis and to evaluate the safety profile of posaconazole in children.

### 2.3. Posaconazole Administration and Therapeutical Drug Level Monitoring

The starting dose of POS for patients 13 years and older was 3 × 200 mg/day. For patients under 13 years of age, doses ranging from 100 to 600 mg/day were used depending on age, body weight and body surface area, as there were no established doses for paediatric patients under 13 years of age during the first half of the observation period. For the DRT and IV formulations, the initial dose varied between 5 and 10 mg/kg twice on the first day and continued thereafter with 5 and 10 mg/kg/day.

We measured the trough concentration of posaconazole in serum (C_min_) using high-performance liquid chromatography (HPLC). Turnaround time was less than 36 h. Pharmacokinetic (PK) samples were taken 7 days after initiation of prophylaxis and then once a week until treatment discontinuation. Similarly, the first PK samples were taken 7 days after a change in formulation, dose, or dosing interval of posaconazole. Therefore, the observed concentrations represent values obtained at or near steady state, as supported by the pharmacokinetic model described by Pena-Lorenzo [17]. We must note that measurement of posaconazole levels was not possible for three months (14/AUG/2017–11/NOV/2017) during the study period due to a technical problem, so we were unable to obtain PK data from this period.

Posaconazole dose was titrated to a target range of 0.7–2.0 mg/L with each formulation based on weekly TDM. The maximum daily dose was 800 mg for POS and 400 mg for the DRT and IV formulations. Posaconazole prophylaxis was administered in neutropenic patients until neutrophil engraftment, and in patients with acute GvHD until the systemic methylprednisolone dose was reduced below 0.5 mg/kg.

### 2.4. Evaluation of C_min_

C_min_ was investigated with a mixed model [18]; the model specification is presented below:

log(C_min,ss,i,j_) = β_0_ + β_1_ × BSA_i_ + β_2_ × Enteral symptoms_i,j_ + β_3_ × Antacid use_i,j_ + β_4_ × Mucositis occurrence_i,j_ + β_5_ × Sex_i_ + β_6_ × Daily dose_i,j_ ∗ β_7,k_ × Formulation + δ_0,i_ + δ_1i_ × j + ϵ_i,j_

where,

**β** represents the coefficients,
**i** is the subject number,
**j** is the week number,
**k** is the formulation,
**δ** the random effects, and
**ϵ** is the residual error.

Enteral symptoms were defined as Grade ≥ 2 diarrhoea or Grade ≥ 2 vomiting, whereas mucositis was defined as Grade ≥ 3 mucositis according to the Common Terminology Criteria for Adverse Events (CTCAE) version 5.0. Both enteral symptoms and mucositis and antacid use were considered positive if they occurred in the week before the measurement of posaconazole plasma levels. The reference level for formulation was set as the DRT. The size of the patient was best taken into consideration using body surface area (BSA), with higher BSA based on model fit.

### 2.5. Statistical Analysis

Analyses were conducted using the R statistical language (version 4.3.1; R Core Team, 2023) on Windows 10 × 64 (build 19045). Mixed modelling was carried out using the package lme4, version 1.1.35.1 [18]. The survival analyses were carried out using the package survival (version 3.5.7; Therneau 2023).

While for time-to-event analyses the sample size and number of events were extremely low, Cox regression was attempted to highlight possible associations between events and variables. A Cox model with Formulation as a single variable minus average log(C_min_) throughout the treatment period per patient was initially fitted, and variable selection was carried out via refitting the original model sequentially with Sex, Age at transplant, Formulation or BSA as a second independent variable. The model with the highest Nagelkerke R2 was then chosen as the best model of interest. Given the limited number of events on which these models were fitted, this procedure would keep the resulting models relatively simple. Also, the results could also be interpreted as a sensitivity analysis, confirming the impact of the C_min_ levels when investigated alone or when controlled for with a variety of variables.

## 3. Results

### 3.1. Patient Characteristics

A total of 113 patients received posaconazole prophylaxis during the study period, 105 of whom the formulation was specified, and 102 of whom had at least one posaconazole therapeutic drug level monitoring (TDM). Patients without TDM were excluded from further analyses. The characteristics of the 102 patients with posaconazole TDM are summarised in Table 1.

### 3.2. Posaconazole Pharmacokinetics

**Figure 1:**
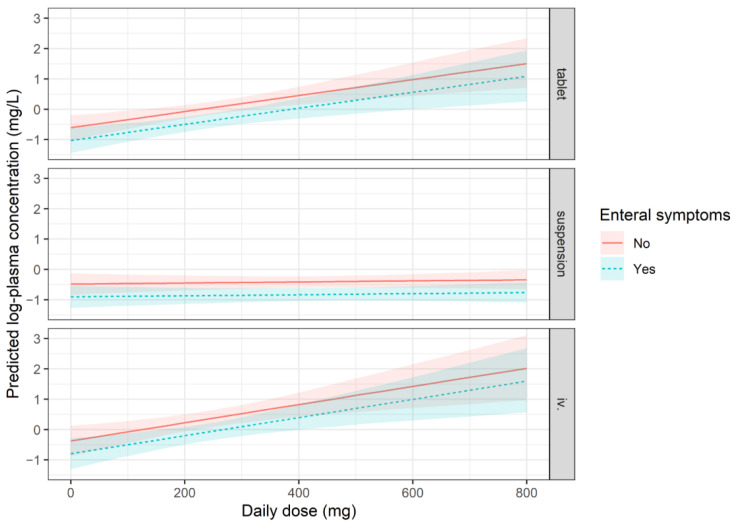
Representation of the expected mean plasma concentrations according to the mixed model. All terms which are in the model have to be taken into account, and the plot therefore supposes patients with 1 m^2^ Body Surface Area, not experiencing mucositis, and are without antacid use. The expected mean values and a 95% confidence band are plotted in two cases; a solid line represents patients with no enteral symptoms, and a dashed line represents patients who experience enteral symptoms. See model specification for C_min_ in Supplementary Materials Table S1.

**Figure A1:**
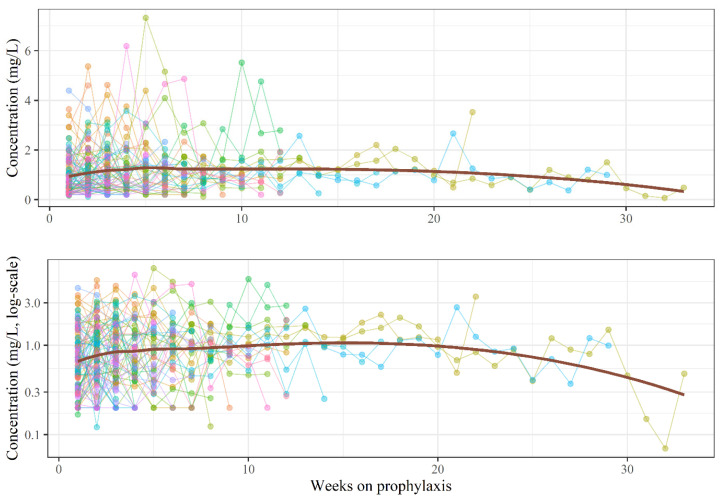
Posaconazole plasma concentrations per week. The upper curve is drawn on linear scale, while the lower curve is on log-scale. Each color represents a different patient, while red line represents a LOESS curve of the mean.

We measured posaconazole plasma concentrations in a total of 548 blood samples, with a median of four measurements per patient (IQR: 2–7). The overall variability (CV, coefficient of variability) of measurements was 85.17%, and the intra-subject variability for patients with at least two measurements was 65.14% (counting measurements of the most frequent formulation per patient if a patient received more than one formulation type during follow-up). No major trends were observed when plasma concentrations were examined as a function of time (Figure A1); active management of administered doses kept patients well controlled. In the event of a dose change, doses were promptly adjusted, generally resulting in normalisation of plasma concentrations after one week. C_trough,ss_ was analysed using a mixed model. The fixed part of the model explains only about 14% of the observed variance, whereas about 50% of the variance is explained when random effects are accounted for. This suggests that the results are highly specific to individual patients. The dose–concentration curve of the suspension formulation is markedly flatter (0.01 elevation in log-concentration for each additional 100 mg posaconazole administered, which corresponds to a ~1% increase in plasma concentration) than that of the DRT or IV formulations, signifying a poor ability to titrate the dosage with this formulation (Figure 1).

According to the model, the appearance of enteral symptoms during prophylaxis was associated with lower plasma concentrations (*p* < 0.001), which could be explained by lower absorption in the presence of symptoms (Figure 1). Simultaneous administration of PPI and posaconazole did not result in significantly lower plasma levels (*p* = 0.095); however, there was a trend-like difference. This difference was found to be similar, and not statistically significant when we refitted the model with an interaction term, analyzing the three formulations separately. We also did not observe any association between mucositis and posaconazole plasma levels.

The mean administered doses of the POS, DRT and IV formulations were 17.2, 7.41, and 7.75 mg/kg, respectively, whereas the doses associated with the target range of 0.7–2.0 mg/L were 15.22, 7.52, and 7.84 mg/kg, respectively (Table 2). Of note, lower plasma concentrations were associated with a higher posaconazole suspension dose (18.57 mg/kg), showing the unpredictability of this formulation.

**Figure 2:**
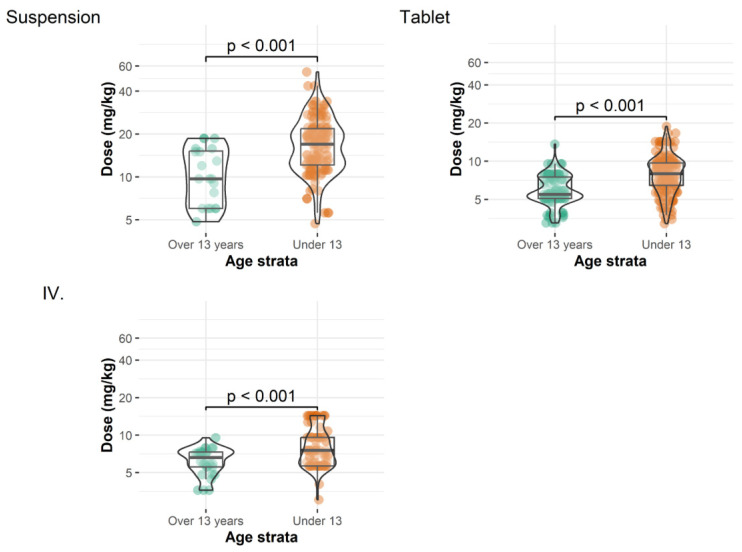
Administered doses of different posaconazole formulations for patients ≥ 13 years and <13 years before each measurement.

Patients less than 13 years old required significantly higher doses (per kilogram body weight) than patients older than 13 years (Table 3, Figure 2). Note that the values presented describe the entire dataset without accounting for the correlated nature of the data (i.e., measurements from the same patient are more closely correlated than those from different patients). Consequently, these results should be interpreted as a general representation of plasma concentration values when active efforts are made to maintain plasma concentrations at a specific level. In general, the use of the IV formulation was associated with the highest proportion of target plasma concentrations between 0.7–2.0 mg/L (65.8% of samples), followed by the DRT (49.4%) and POS (33.3%) formulations.

### 3.3. Breakthrough Infections and Mortality

**Figure 3:**
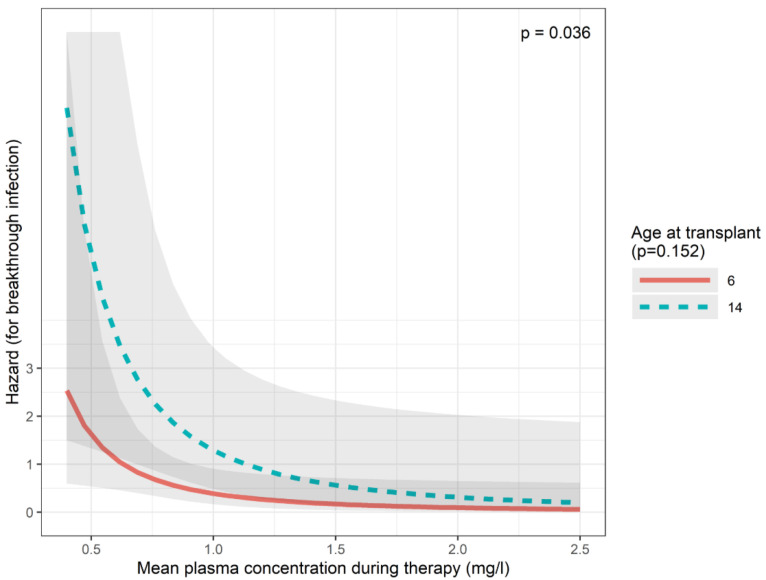
Predictions of the breakthrough infection (Cox) model.

Based on the definitions of the European Organization for Research and Treatment of Cancer and the Mycoses Study Group [19], we observed one proven and seven probable invasive fungal breakthrough infections (BIs) during the study period (7.8%). The proven case was a disseminated infection with *Fusarium proliferatum* diagnosed by both culture and histology. All seven probable cases were pulmonary infections with either galactomannan positivity or positive culture from bronchoalveolar lavage. A posaconazole-resistant fungus was detected in only 1/8 cases, whereas low levels of posaconazole (<0.7 mg/L) were observed in 5/8 BIs. All BI events occurred in males, and the effect of sex is therefore not directly evaluable with a Cox model. The Cox regression model showed that an increase in average log-plasma concentration decreased the hazard of a BI (*p* = 0.036 for the best model including plasma concentration and age at transplant). This effect was robust for including alternative variables instead of age at transplant (such as formulation, body surface area, or omitting a second variable altogether). Compared to the plasma concentration of 0.7 mg/L (HR: 1), the HR for BI at 1.2 mg/L would be 0.33, at 1.5 mg/L, it would be 0.21, and at 2.0 mg/L, it would be 0.12 according to the best model (Figure 3, Table A1).

**Figure A2:**
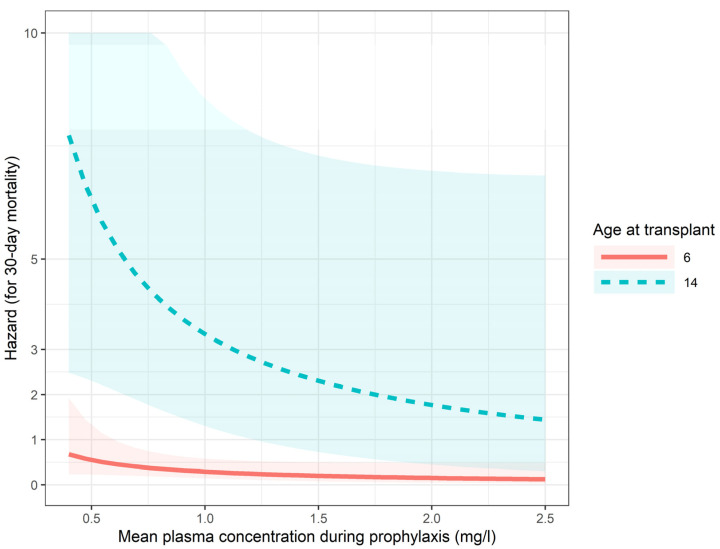
Predictions of the 30-day mortality (Cox) model.

All-cause mortality at 30 days post prophylaxis was defined as death within 30 days after posaconazole discontinuation. A total of 11 patients (10.8%) died within 30 days after discontinuation of prophylaxis: 10 cases were considered transplant-related mortality and one relapse. The role of mean plasma concentration during prophylaxis in mortality was examined with Cox models (Figure A2, Table A2). According to the best model in terms of Nagelkerke R^2^, higher age at transplantation was associated with a higher risk of death (*p* = 0.002). The effect of higher posaconazole levels while seems protective (HR 0.40, CI: 0.14–1.17) was not statistically significant (*p* = 0.094).

### 3.4. Safety

We investigated the association between plasma levels of posaconazole and the elevation of liver enzymes. Elevated blood alanine aminotransferase (ALAT), aspartate aminotransferase (ASAT), alkaline phosphatase (ALP), gamma-glutamyl transferase (GGT), and bilirubin levels were measured and classified into four categories (grade I–IV) according to the CTCAE version 5.0. Grade III–IV elevation of any of the above liver parameters was noted in 18 patients. In the physician’s judgement, 15 of these events were considered part of the underlying disease, graft-versus-host disease, or a side effect of conditioning and resolved spontaneously, whereas posaconazole was discontinued in three cases in which drug toxicity was assumed.

**Figure A3:**
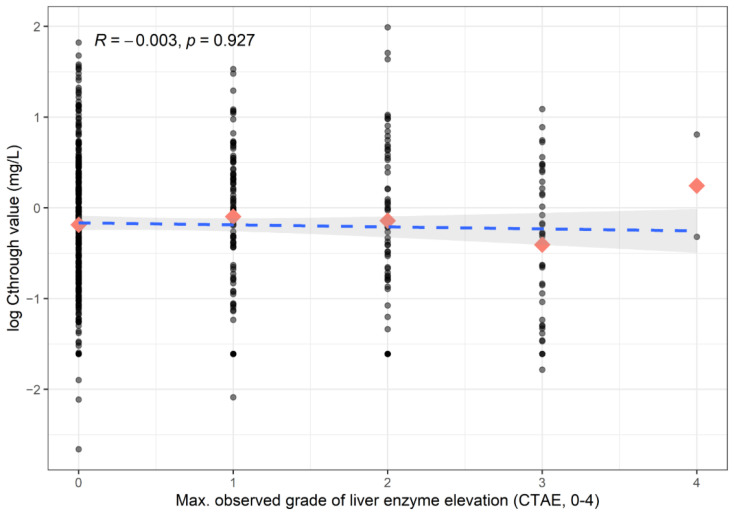
Association between liver enzyme elevation and posaconazole levels. Dashed line represents the Kendall-correlation. Rectangles represent the mean values per category.

In the 12 patients who had posaconazole levels > 4.0 mg/L, we did not observe any Grade III–IV elevation of liver enzymes. We also did not demonstrate any association between the elevation of liver enzymes and posaconazole serum levels (Figure A3).

In 42 patients (41.2%), we observed hypertension requiring antihypertensive therapy. Although these patients received a large number of concomitant medications, only 23 (55%) were treated with corticosteroids or other compounds that could be held responsible for the elevated blood pressure. We analysed the association between posaconazole levels and the occurrence of hypertension using generalised mixed modelling, but we did not find a statistically significant correlation between these variables (*p* = 0.282).

Of note, hypertension did not lead to complications in our patient population. In addition, we did not observe any severe cardiac arrhythmias, torsades de pointes, or polymorphic ventricular tachycardia during the study period.

## 4. Discussion

We conducted a retrospective study to obtain real-world data on the pharmacokinetics, efficacy, and safety of prophylactic posaconazole in paediatric allo-HSCT patients. For POS, the daily dose required to achieve target exposure was 15.2 mg/kg, quite similar to previously published data in paediatric patients from different geographic areas [20,21]. The DTR and IV formulations have better bioavailability, so lower doses are required for the same drug exposure. The reported starting doses, which were mostly used, ranged from 5–10 mg/kg/day, and a loading dose (the maintenance dose given twice on the first day) is suggested [7,22,23]. In our patients, the daily doses required to reach target levels were well within this range: 7.5 and 7.8 mg/kg for the DRT and IV formulations, respectively. A recent study suggested that paediatric patients under 12 years old needed higher doses of posaconazole to achieve therapeutic range [21]. Likewise, we found that paediatric patients under 13 years old required significantly higher doses of the POS and DRT formulations compared to patients older than 13 years (POS 13.0 vs. 6.0 mg/kg and DRT 7.9 vs. 5.9 mg/kg, respectively).

Pharmacokinetics and oral absorption of posaconazole are influenced by a multitude of factors. Studies in healthy individuals have shown that a high-fat meal and in-meal and post-meal administration increase the absorption of posaconazole, whereas higher gastric pH and increased gastric motility have a negative effect [5]. Indeed, gastric conditions, like proton pump inhibitor administration, vomiting, diarrhoea, and inability to eat and swallow due to gastrointestinal mucositis, are common problems in stem cell transplant recipients and patients with hematologic malignancies. Not surprisingly, posaconazole suspension has shown unreliable absorption rates and exposures in real-world studies [24]. In our study, the dose–concentration curve of the suspension formulation was significantly flatter than that of the tablet or intravenous formulation, suggesting that increasing the dose of the suspension formulation may not result in higher posaconazole levels. In addition, we found that when POS was used, lower plasma concentrations were associated with a higher dose, which also demonstrates the poor predictability of this formulation. Considering that 7 days are needed to reach steady-state conditions and reassess posaconazole exposure after dose escalation, a patient with consecutive 2 or 3 under-the-target drug levels will have suboptimal exposure and will therefore not be protected from fungal infections for weeks. By using the DRT and IV formulations, however, some of these issues can be overcome, allowing consistent, once-daily dosing [7,8]. In our paediatric real-world study, similarly to published data, we found that the DRT and especially the IV formulation outperformed the POS in terms of predictability and consistency. It should be noted that in 2021, the FDA approved a new posaconazole formulation, a delayed-release oral suspension possibly combining the advantages of POS and DRT formulations. In our study, no pharmacokinetic data could be determined for this formulation due to limited drug availability.

In our study, we found that diarrhoea negatively influences posaconazole plasma levels. This is a well-described effect, and is mostly explained by poor gastrointestinal absorption. However, in one patient in our cohort who suffered from Grade 3 diarrhoea, we were unable to reach target plasma levels even with high doses (11 mg/kg) of IV formulations. This raises the question if poor absorption is the only factor in reducing drug levels in patients with diarrhoea. Another possible explanation could be the increased gastrointestinal excretion through the inflamed mucosa, as approximately 66% of the total dose is excreted as an unchanged drug with faeces.

Increased gastric pH and the concomitant use of PPIs are known to reduce absorption when using POS formulation [5,20]. However, with the DRT formulation, this effect seems to be diminished [25]. In our study, we found that coadministration with PPI did not significantly influence the C_min_ even if the three formulations were separately analysed. The lack of negative effects of PPI on drug levels with the POS might be explained by the low dose and the short duration of PPI prophylaxis.

Posaconazole generally has a favourable safety profile. Triazole administration can lead to both hepatocellular and cholestatic liver injury, but the mechanism of toxicity is not well understood. An in vitro study has shown that posaconazole can lead to mitochondrial dysfunction and induce apoptosis, suggesting that preexisting mitochondrial dysfunction is a susceptibility factor for hepatotoxicity [26]. Genetic factors may also play a role in posaconazole toxicity, particularly in the rare cases of idiosyncratic drug-induced liver injury [27]. To date, no threshold for toxicity of posaconazole serum levels has been established. In fact, toxicity does not appear to be related to elevated drug levels in a broad therapeutic window [28]. We examined both hepatocellular and cholestatic toxicity in our study and found a very low rate of suspected posaconazole toxicity. We also found no association between the elevation of liver enzymes and posaconazole serum levels, which is consistent with the literature. In patients with very high posaconazole exposure (>4 mg/L), we observed no Grade III–IV liver toxicity. These data suggest that posaconazole is a safe antifungal drug, but in the rare cases of some patient-related factors, toxicity can be expected even without high drug exposure.

Hypertension is a known side effect of posaconazole, but there are few real-world data on its incidence. Elevated blood pressure may be related to pseudohyperaldosteronism [29]. To date, several case reports addressed the possible underlying mechanism, and two different pathways have been found: inhibition of 11β-hydroxylase and 11β-hydroxysteroid dehydrogenase 2 [30,31,32]. Because deficiency of 11β-hydroxylase is the hallmark of congenital adrenal hyperplasia, posaconazole can induce a similar clinical and biochemical picture [33]. The factors responsible for the differential inhibition of these enzymes are not fully understood. Significant interindividual differences have been described, possibly related to genetic polymorphisms or other factors affecting the pharmacokinetics of posaconazole. Recently, posaconazole-induced pseudohyperaldosteronism was reported in 23.2% of treated patients, suggesting that its development is associated with higher serum concentration, older age, and baseline hypertension [34]. To our knowledge, this is the first study to report a high rate of hypertension as a possible side effect of posaconazole in paediatric allo-HSCT recipients. We found hypertension in an unexpectedly high number of patients (41.2%), and almost half of them were found to have no other obvious cause of hypertension. Using generalised mixed modelling, we observed only a weak relationship between posaconazole plasma concentration and hypertension, which did not reach statistical significance. Of note, the model suffered from a poor fit, as evidenced by the distribution of the random effects, which suggests the role of other major independent variables responsible for the occurrence of hypertension. However, based on our results, attention should be paid to hypertension in addition to hepatic toxicity in paediatric patients treated with posaconazole.

Eight BIs were identified in the study population (7.8%), which seems somewhat higher compared to reports from the literature [35,36,37,38]. However, these data differ in terms of patient age, underlying disease, and definitions of breakthrough infections (i.e., whether possible infections were included or not). There are limited data on BIs in paediatric patients on posaconazole prophylaxis. In a recent study including only paediatric patients with hematologic disorders, a similar rate of breakthroughs was reported [39]. In the present patient cohort, five patients with breakthrough infections had low plasma posaconazole concentrations at the time of diagnosis, whereas only one patient had a biopsy- and culture-confirmed posaconazole-resistant fungal infection (*Fusarium proliferatus*). Results also suggest an exposure–response relationship, underscoring the importance of TDM in paediatric patients.

In our study, we found that higher posaconazole concentration significantly reduced the hazard of BIs. This is in contrast with results from a meta-analysis aiming to evaluate the exposure–response relationship of posaconazole. Data from this analysis suggest the optimal target posaconazole concentration being 0.5 mg/L instead of the more widely used 0.7 mg/L for the prevention of invasive fungal infections, because no clear benefit was evident for the latter [15]. However, the underlying disease of the patients who participated in this meta-analysis was heterogeneous not only including HSCT cases but also patients with hematologic malignancies, cardiothoracic transplants, and chronic granulomatosus disease. On the other hand, there are numerous studies supporting an exposure–response relationship for posaconazole prophylaxis. Here, favourable results were mostly observed in cases with posaconazole serum concentrations above 0.5–0.7 mg/L [37,40,41,42]. Moreover, in a recent study in allo-HSCT patients on posaconazole prophylaxis, a low number of BI was observed possibly explained by the higher overall posaconazole level (1.3 mg/L) [43].

When examining the efficacy of posaconazole prophylaxis, the changing epidemiology of invasive fungal infections should also be considered. With the increasing number of patients with prolonged and profound immunosuppression and the widespread use of anti-Aspergillus prophylaxis, higher rates of mucormycosis, fusariosis cases, and multidrug-resistant fungal infections have been reported [44,45]. It is not yet clear whether these relatively rare mould infections can be more successfully prevented with higher posaconazole exposure. Therefore, we cannot recommend higher target values for prophylaxis, as the proof from our data is not sufficient. Further studies should be performed to confirm the optimal posaconazole plasma level for prophylaxis.

Our study has several limitations in addition to its retrospective nature. First, due to a technical problem, posaconazole levels could not be measured for three months. Second, when we analysed the C_min_ levels with a mixed model, the fixed part explained only about 14% of the observed variance, whereas about 50% of the variance was explained when random effects were accounted for. This suggests that the results are very specific to individual patients and that we might have other, not observed confounding factors that influence the results. Third, the main objective of this study was to provide pharmacokinetic data and not to analyse risk factors for BIs and mortality. We used the Cox model to investigate time-to-event data. Our results, suggesting a correlation between posaconazole serum levels and Bis, seem persuasive; however, this should be viewed with scepticism due to the very few observed events (for BIs and mortality), and the fact that the analysis relies heavily on the condition that mean plasma levels during therapy are independent from the length of prophylaxis. Although we refitted these models with several variables along with plasma concentration partly to provide persuasive sensitivity analyses, the results should be evaluated as preliminary. Also, we did not account for major risk factors, such as duration of neutropenia and corticosteroid treatment; however, all patients enrolled in this study were at high risk for IFI, either because of neutropenia after HSCT or GvHD treated with corticosteroids. Despite these limitations, we believe that our real-world data will improve knowledge of the pharmacokinetics, efficacy, and safety of posaconazole in paediatric patients.

## 5. Conclusions

Posaconazole doses of 15.2, 7.5, and 7.8 mg/kg for POS, DRT, and IV formulations were associated with the target range between 0.7–2.0 mg/L. With the DRT formulation, patients under 13 years old needed higher doses to achieve the target range (7.9 mg/kg vs. 5.9 mg/kg). The DRT and IV formulations outperformed the POS in terms of predictability. Posaconazole was generally well tolerated in our study. We detected hypertension in an unexpectedly high rate of patients; however, many confounding factors were present. Our analyses on BIs and posaconazole plasma levels suggest an exposure–response relationship. Based on our data, targeting higher posaconazole plasma levels for prophylaxis is safe and could result in reduced hazard for BIs.

## Figures and Tables

**Table 1:** Patient characteristics.

Characteristic	Overall *n* = 102 ^1^	Suspension *n* = 42 ^1^	Tablet *n* = 47 ^1^	Intravenous *n* = 13 ^1^	*p*-Value ^2^
Age at transplant (years)	9.4 (5.9, 13.0)	5.9 (2.7, 10.1)	11.6 (8.7, 14.2)	8.5 (5.3, 11.9)	<0.001
Mass (kg)	30 (21, 43)	23 (13, 32)	35 (29, 53)	25 (17, 53)	<0.001
Sex (female)	30 (29%)	9 (21%)	15 (32%)	6 (46%)	0.2
Height (cm)	134 (111, 156)	116 (91, 140)	144 (130, 162)	122 (110, 168)	<0.001
BSA	1.04 (0.80, 1.36)	0.86 (0.55, 1.13)	1.15 (1.03, 1.60)	0.91 (0.72, 1.60)	<0.001
Primary disease category					0.5
ALL	23 (23%)	7 (17%)	12 (26%)	4 (31%)	
AML	14 (14%)	6 (14%)	6 (13%)	2 (15%)	
Other Malignancy	14 (14%)	6 (14%)	5 (11%)	3 (23%)	
MDS	28 (27%)	9 (21%)	17 (36%)	2 (15%)	
Severe aplastic anemia	8 (7.8%)	4 (9.5%)	4 (8.5%)	0 (0%)	
Congenital defect	14 (14%)	9 (21%)	3 (6.4%)	2 (15%)	
Other	1 (1.0%)	1 (2.4%)	0 (0%)	0 (0%)	
Myeloablative conditioning	69 (68%)	27 (64%)	32 (68%)	10 (77%)	0.7
Serotherapy	84 (82%)	34 (81%)	37 (79%)	13 (100%)	0.2
Donor type					0.11
Matched family donor	3 (2.9%)	1 (2.4%)	2 (4.3%)	0 (0%)	
Haploidentical donor	13 (13%)	2 (4.8%)	8 (17%)	3 (23%)	
Unrelated donor	64 (63%)	28 (67%)	26 (55%)	10 (77%)	
Matched sibling donor	22 (22%)	11 (26%)	11 (23%)	0 (0%)	
Type of graft					0.3
Bone marrow	65 (64%)	30 (71%)	29 (62%)	6 (46%)	
Cord blood	6 (5.9%)	2 (4.8%)	2 (4.3%)	2 (15%)	
Peripheral blood	31 (30%)	10 (24%)	16 (34%)	5 (38%)	
Indication					0.034
Acute GvHD	13 (13%)	9 (21%)	2 (4.3%)	2 (15%)	
Neutropenia and aGvHD	12 (12%)	3 (7.1%)	9 (19%)	0 (0%)	
Neutropenia post HSCT	70 (69%)	25 (60%)	34 (72%)	11 (85%)	
Other	7 (6.9%)	5 (12%)	2 (4.3%)	0 (0%)	

^1^ Median (IQR); *n* (%); ^2^ Kruskal–Wallis rank sum test; Fisher’s exact test; BSA: body surface area; ALL: acute lymphoblastic leukemia; AML: acute myeloid leukemia; MDS: myelodysplastic syndrome; GvHD: Graft-versus-host disease; aGvHD: acute Graft-versus-host disease; HSCT: hematopoietic stem cell transplantation.

**Table 2:** Administered doses of posaconazole (mg/kg) per observed plasma concentration categories.

Plasma Concentration Category	Suspension *n* = 166 ^1^	Tablet *n* = 255 ^1^	Intravenous *n* = 111 ^1^	*p*-Value ^2^
low (<0.7 mg/L)	18.62 (6.60)	6.98 (2.76)	6.85 (1.70)	
medium (0.7–2 mg/L)	15.22 (8.70)	7.52 (2.62)	7.84 (2.84)	
high (>2 mg/L)	17.09 (10.18)	8.27 (2.54)	9.37 (4.16)	
Overall dose	17.23 (8.12)	7.41 (2.68)	7.75 (2.83)	0.002

^1^ Mean dose, mg/kg (SD); ^2^ Kruskal–Wallis Rank Sum Test.

**Table 3:** Administered posaconazole dose with different formulations and plasma level categories achieved in patients ≥ 13 years and <13 years.

	Low (<0.7 mg/L)			Medium (0.7–2 mg/L)			High (>2 mg/L)		
Formulation	≥13 Years (*n* = 61 ^1^)	<13 Years (*n* = 144 ^1^)	*p*-Value ^2^	≥13 Years (*n* = 80 ^1^)	<13 Years (*n* = 174 ^1^)	*p*-Value ^2^	≥13 Years (*n* = 12 ^1^)	<13 Years (*n* = 61 ^1^)	*p*-Value ^2^
Suspension	16 (3.2)	19 (8.9)	0.2	6.0 (3.7)	13 (8.5)	<0.001	9.3 (1.8)	14 (8.9)	0.017
Tablet	5.4 (2.1)	6.9 (3.7)	<0.001	5.9 (2.8)	7.9 (3.0)	<0.001	5.9 (1.4)	9.7 (2.9)	0.018
Intravenous	5.7 (1.8)	7.2 (2.0)	0.042	7.1 (1.9)	7.5 (3.9)	0.055	6.1 (0.00)	8.6 (8.1)	0.6

^1^ Median dose, mg/kg (SD); ^2^ Wilcoxon Rank Sum Test.

## Data Availability

The raw data supporting the conclusions of this article will be made available by the authors upon request.

## Supplementary Materials

The following supporting information can be downloaded at: https://www.mdpi.com/article/10.3390/children12040467/s1, Table S1: Model specification for C_min_ used in Figure 1.

## Appendix A

**Table A1:** Hazard ratio (HR) change for breakthrough infection according to changes of mean plasma concentration. Initial and follow-up values as rows and columns, respectively, e.g., an increase in plasma concentration from 0.7 mg/L to 1.2 mg/L would result in a reduction in the HR to 33% (0.12–0.93).

Plasma Concentration	0.7 mg/L	1.2 mg/L	1.5 mg/L	2 mg/L
0.7 mg/L	HR: 1 (1–1)	HR: 0.33 (0.12–0.93)	HR: 0.21 (0.05–0.9)	HR: 0.12 (0.02–0.87)
1.2 mg/L	HR: 3.02 (8.45–1.08)	HR: 1 (1–1)	HR: 0.63 (0.41–0.97)	HR: 0.35 (0.13–0.93)
1.5 mg/L	HR: 4.76 (20.45–1.11)	HR: 1.58 (2.42–1.03)	HR: 1 (1–1)	HR: 0.55 (0.32–0.96)
2 mg/L	HR: 8.59 (63.89–1.15)	HR: 2.85 (7.56–1.07)	HR: 1.8 (3.12–1.04)	HR: 1 (1–1)

**Table A2:** Hazard ratio (HR) change for mortality at 30 days post prophylaxis according to changes in mean plasma concentration. Initial and follow-up values as rows and columns, respectively, e.g., a reduction of plasma concentration from 1.5 mg/L to 0.7 mg/L would result in an increase in the HR to 201% (0.88–4.58).

Plasma Concentration	0.7 mg/L	1.2 mg/L	1.5 mg/L	2 mg/L
0.7 mg/L	HR: 1 (1–1)	HR: 0.61 (0.34–1.09)	HR: 0.5 (0.22–1.13)	HR: 0.38 (0.12–1.18)
1.2 mg/L	HR: 1.64 (2.93–0.92)	HR: 1 (1–1)	HR: 0.81 (0.64–1.04)	HR: 0.63 (0.36–1.09)
1.5 mg/L	HR: 2.01 (4.58–0.88)	HR: 1.23 (1.56–0.96)	HR: 1 (1–1)	HR: 0.77 (0.56–1.05)
2 mg/L	HR: 2.62 (8.13–0.84)	HR: 1.6 (2.77–0.92)	HR: 1.3 (1.78–0.95)	HR: 1 (1–1)
