# Supplementary material for: Real World Posaconazole Pharmacokinetic Data in Paediatric Stem Cell Transplant Recipients
Source: Children (Basel). 2025 Apr 5;12(4):467. doi: 10.3390/children12040467 (PMC12026224; doi:10.3390/children12040467)
Supplement: Supplementary file 1 [file children-12-00467-s001.zip › children-3528970-supplementary.pdf]

Supplementary Materials

Table S1. Model specification for C_{min} used in Figure 1.

	log(C_{min})		
**Predictors**	**Estimates**	**Confidence interval**	***p***
(Intercept)	-0.06	-0.59–0.47	0.817
Body Surface Area	-0.55	-0.89–-0.21	**0.002**
Enteral symptoms	-0.42	-0.58–-0.27	**<0.001**
Antacid use	-0.16	-0.35–0.04	0.109
Formulation: suspension	0.14	-0.37–0.66	0.591
Formulation: intravenous	0.21	-0.31–0.74	0.418
Mucositis	-0.08	-0.29–0.13	0.451
Sex (female)	0.22	-0.05–0.49	0.107
Daily dose (per 100 mg)	0.27	0.12–0.42	**<0.001**
Antacid x Formulation: Suspension	0.01	-0.35–0.37	0.944
Antacid x Formulation: intravenous	0.13	-0.22–0.47	0.477
Formulation: suspension x Daily dose (per 100 mg)	-0.26	-0.41–-0.10	**0.002**
Formulation: intravenous x Daily dose (per 100 mg)	0.01	-0.19–0.22	0.922
**Random Effects**			
σ^2	0.38		
$\tau_{00\ ID}$	0.26		
$\tau_{11\ ID.week\_no}$	0.00		
ρ_{01}			
ρ_{01}			
ICC	0.40		
N_{ID}	101		
Observations	540		
Marginal R^2/Conditional R^2	0.144/0.488		
